# Establishment of a Thioacetamide-Induced Hepatotoxicity Model in Synanthropic Rats with Translational Relevance

**DOI:** 10.3390/diseases14040142

**Published:** 2026-04-11

**Authors:** Lesly Adelis Valdivia Quispe, Lucio Velasco Lopez, Daysi Zulema Díaz Obregón, Alexis German Murillo Carrasco, Joel de León Delgado, Luis Lloja Lozano, Jhon Wilfredo Pando Mayta, Anthony Brayan Rivera Prado, Kelly Geraldine Yparraguirre Salcedo, Víctor Hugo Carbajal Zegarra, Claudio Willbert Ramírez Atencio

**Affiliations:** 1 Laboratory of Regenerative Medicine, Universidad Nacional Jorge Basadre Grohmann, Tacna 23003, Peru; lvaldiviaq@unjbg.edu.pe (L.A.V.Q.); lvelascol@unjbg.edu.pe (L.V.L.); lllojal@unjbg.edu.pe (L.L.L.); ariverap@unjbg.edu.pe (A.B.R.P.); kyparraguirres@unjbg.edu.pe (K.G.Y.S.); vcarbajalz@unjbg.edu.pe (V.H.C.Z.); 2 Innovation and Science for the Care and Support of Society—INNOVACARE, Lima 15024, Peru; daysi.diaz@essalud.gob.pe; 3 Immunology and Cancer Research Group (IMMUCA), OMICS, Lima 15001, Peru; agmurilloc@usp.br; 4 Faculty of Medicine, Research Center for Virology, Universidad de San Martín de Porres, Lima 15001, Peru; jdeleond@usmp.pe; 5 Institute of Cryopreservation and Cell Therapy, Lima 15001, Peru; jpando@criocord.com.pe

**Keywords:** hepatotoxicity, thioacetamide, synanthropic rats, liver injury, experimental liver disease, preclinical model

## Abstract

**Background/Objectives**: Chemically induced hepatotoxicity is widely used in experimental research to model liver disease pathophysiology and to support preclinical studies. Thioacetamide (TAA) is a well-established hepatotoxic agent in conventional laboratory rodents; however, its effects in synanthropic rats—characterized by genetic heterogeneity and chronic environmental exposure—remain poorly defined. This study aimed to establish and characterize a preclinical model of TAA-induced hepatotoxicity in synanthropic rats and to assess its relevance for experimental liver disease research. **Methods**: Female synanthropic rats representing four phenotypic variants (albino, mottled, black, and brown; total *n* = 132) were housed under controlled conditions and assigned to control or TAA-treated groups. TAA was administered intraperitoneally at doses ranging from 200 to 300 mg/kg. Clinical parameters, including body weight and vital signs, were periodically monitored. Hematological profiles and serum biochemical markers of liver function were analyzed. Hepatic injury was evaluated by histopathological examination using hematoxylin–eosin staining. Statistical analyses were performed using R software, with *p* ≤ 0.05 considered statistically significant. **Results**: TAA-treated rats developed consistent clinical manifestations of hepatotoxicity, including progressive weight loss and reduced activity. Biochemical analyses revealed significant increases in serum transaminases, gamma-glutamyl transferase, and alkaline phosphatase, accompanied by alterations in hematological parameters. Histological evaluation demonstrated dose-dependent liver injury characterized by centrilobular necrosis, inflammatory infiltration, hepatocellular degeneration, and architectural disruption across all synanthropic rat variants. **Conclusions**: Synanthropic rats exhibit reproducible biochemical, hematological, and histopathological features of TAA-induced liver injury comparable to those reported in conventional laboratory strains. This model represents a robust preclinical approach for studying chemically induced hepatotoxicity and may provide enhanced translational relevance due to its genetic and environmental heterogeneity.

## 1. Introduction

Chemically induced hepatotoxicity has been extensively explored in experimental models due to its relevance for elucidating the pathophysiological mechanisms underlying human liver diseases and for evaluating the safety of pharmacological and environmental compounds [1]. Among the hepatotoxic agents commonly employed, thioacetamide (TAA) is a well-established compound that induces reproducible liver injury through metabolic activation, leading to oxidative stress, inflammatory signaling, and hepatocyte apoptosis. Because of these characteristics, TAA-based models have been widely used to investigate the molecular and cellular mechanisms driving hepatic injury and disease progression under controlled experimental conditions [2].

Experimental models of chemically induced liver injury remain a cornerstone of preclinical research, as they provide a controlled framework for studying toxicological mechanisms and for evaluating potential therapeutic strategies in hepatology [3]. In particular, TAA-induced models have been extensively applied not only to characterize liver damage and fibrogenesis but also as experimental platforms for testing antifibrotic and regenerative approaches [4].

Most studies on TAA-induced hepatotoxicity have traditionally relied on conventional laboratory rat strains, such as Wistar or Sprague–Dawley, owing to their genetic homogeneity and standardized breeding conditions [5]. While these models offer high experimental reproducibility, their reduced biological variability may limit external validity and fail to fully reflect the heterogeneity observed in human liver disease. This limitation has prompted increasing interest in alternative experimental models that better capture the influence of genetic diversity and environmental exposure on hepatic responses [6].

Synanthropic rats (*Rattus norvegicus*), which coexist closely with humans in urban environments, are chronically exposed to a wide range of environmental stressors, including chemical pollutants, nutritional variability, and psychosocial stress. This sustained exposure, together with their intrinsic genetic heterogeneity, may influence hepatic susceptibility to toxic injury and modulate disease progression. Despite their ecological relevance and potential translational value, the hepatic response of synanthropic rats to chemically induced hepatotoxicity, particularly following TAA exposure, has not been comprehensively characterized [7].

Evidence from experimental toxicology indicates that genetic diversity and environmental context play a critical role in shaping hepatic responses to toxic insults and represent key determinants of external validity in preclinical research [8]. In this context, evaluating TAA-induced liver injury in synanthropic rats may provide valuable insights into hepatotoxic mechanisms under environmentally relevant conditions and support the validation of these animals as alternative preclinical models for hepatological and toxicological research.

In the present study, we evaluated the susceptibility of synanthropic rats to TAA-induced hepatotoxicity by analyzing clinical parameters, hematological and biochemical alterations, and histopathological changes. By characterizing hepatic responses in genetically heterogeneous synanthropic rat populations, this work aims to establish a reproducible preclinical model with potential translational relevance for the study of liver disease pathophysiology and for future applications in experimental toxicology and therapeutic research.

In recent years, the translational relevance of preclinical animal models has been increasingly examined, particularly regarding external validity and the generalizability of findings derived from genetically homogeneous laboratory strains [9,10]. Comparative analyses have highlighted that the translational gap between animal models and human disease may partially reflect limited biological variability under controlled laboratory conditions [11,12]. Importantly, biological heterogeneity should not be regarded merely as experimental noise but rather as an informative dimension of physiological response [13]. Studies have also demonstrated that phenotypic variation in outbred populations does not inherently compromise reproducibility when experimental conditions are appropriately standardized [14]. In this context, evaluating toxicological responses in synanthropic rat populations—characterized by natural genetic diversity and environmental adaptation—may provide complementary insight into the reproducibility and biological consistency of chemically induced liver injury under heterogeneous conditions.

Although the primary focus of this study was hepatic injury, selected physiological parameters, including heart rate and respiratory rate, were monitored throughout the experimental period to provide an integrative assessment of systemic responses during toxic exposure. These measures were incorporated as part of comprehensive physiological monitoring and were not intended to constitute independent experimental endpoints.

The objective of this study was not to compare multiple hepatotoxic agents, but to evaluate whether synanthropic rats, characterized by greater genetic and environmental heterogeneity, develop reproducible hepatotoxic injury when exposed to a well-established chemical model. Thioacetamide (TAA) was selected due to its extensive validation and reproducible dose-dependent hepatic effects in conventional laboratory strains, allowing controlled translational assessment in an alternative rat population.

## 2. Materials and Methods

### 2.1. Experimental Animals

Domesticated synanthropic rats (*Rattus norvegicus*) were used in this experimental study. At the start of the experimental procedures, animals were approximately three months old and exhibited comparable physiological conditions.

Animals were housed in the animal facility of the Faculty of Sciences, Universidad Nacional Jorge Basadre Grohmann (UNJBG), Tacna, Peru, under controlled environmental conditions, including a 12 h light/dark cycle, ambient temperature maintained between 18 and 22 °C, relative humidity of 55–65%, and adequate ventilation (10–15 air changes per hour). Throughout the experimental period, animals had free access to water (*ad libitum*) and were provided with a nutritionally balanced diet once daily.

**Figure 1:**
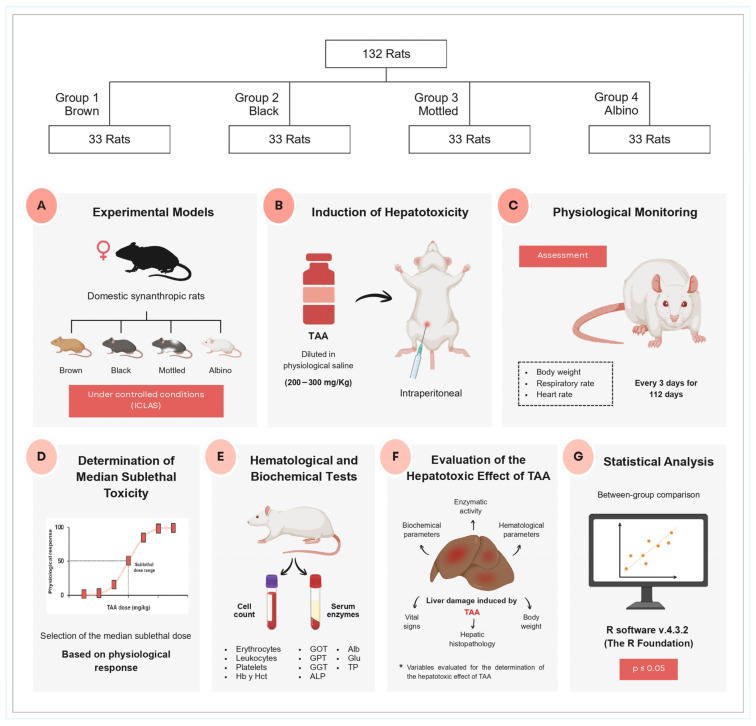
Experimental design and workflow for TAA-induced hepatotoxicity assessment in synanthropic rats. Schematic representation of the study design. A total of 132 female domesticated synanthropic rats (*Rattus norvegicus*) were classified into four phenotypic variants (brown, black, mottled, and albino; *n* = 33 per group) and maintained under controlled laboratory conditions. (**A**) Experimental model showing the four phenotypic variants of synanthropic rats maintained under controlled animal laboratory conditions (CLAS). (**B**) Induction of hepatotoxicity by intraperitoneal administration of thioacetamide (TAA) diluted in physiological saline at doses ranging from 200 to 300 mg/kg body weight. (**C**) Physiological monitoring of animals during the experimental period, including body weight, respiratory rate, and heart rate measurements recorded every three days for 112 days. (**D**) Determination of the median sublethal dose based on physiological response curves. (**E**) Hematological and biochemical analyses performed using blood samples to determine erythrocyte counts, leukocytes, platelets, hemoglobin, and hematocrit, as well as serum biochemical parameters. (**F**) Evaluation of hepatotoxic effects through biochemical markers and liver damage indicators. (**G**) Statistical analysis of experimental data using R software v4.3.2 (v4.3.2, the R Foundation for Statistical Computing, Vienna, Austria), with significance established at *p* ≤ 0.05.

The overall experimental design, including animal grouping, hepatotoxicity induction with thioacetamide (TAA), physiological monitoring, determination of the median sublethal dose, and subsequent hematological and biochemical evaluations, is summarized in Figure 1.

The study was conducted exclusively in female synanthropic rats. The selection of females was based on methodological considerations commonly applied in experimental hepatotoxicity research, including reduced inter-individual aggression, improved group housing stability, and lower stress-induced variability during prolonged experimental protocols. These factors contribute to minimizing confounding behavioral influences on inflammatory and metabolic responses. The primary objective of the present study was to establish and characterize a reproducible preclinical hepatotoxicity model in synanthropic rats rather than to evaluate sex-dependent variability.

A total of 132 female synanthropic rats were included in the study, comprising four phenotypic variants: albino, mottled, black, and brown. Female rats were selected according to the experimental protocol, as their use has been widely reported in chemically induced liver injury models, including thioacetamide-based protocols, showing consistent and reproducible hepatic responses under controlled conditions. Estrous cycle synchronization was not performed.

Animals were distributed into 11 experimental groups according to the established treatment scheme. Prior to the initiation of experimental procedures, all animals underwent an acclimatization period to the housing environment. During the study, animals were routinely monitored for general health status, behavior, body weight changes, and potential signs of distress associated with hepatotoxic injury.

All procedures related to animal housing, handling, monitoring, and experimental manipulation were conducted following standardized protocols commonly applied in preclinical research, with all reasonable efforts made to minimize animal discomfort and ensure reliable experimental outcomes.

### 2.2. Ethical Approval

All experimental procedures involving animals constituted the first stage of a larger research project and were reviewed and approved by the Institutional Ethics Committee of the Hipólito Unanue Hospital of Tacna in November 2022. Ethical approval was granted through a Directoral Resolution (Approval Code: 841-2022-OEG-DRRHH-DR/DRS.T/GOB.REG.TACNA, issued on 25 November 2022).

The experimental protocol was conducted in accordance with national regulations and internationally accepted guidelines governing the ethical use of animals in biomedical research, including the *Guide for the Care and Use of Laboratory Animals* [15] and the *International Guiding Principles for Biomedical Research Involving Animals* [16].

All animal handling and experimental procedures were designed to ensure appropriate animal welfare throughout the study. Animal health status was monitored regularly, and all reasonable efforts were made to minimize suffering and distress while ensuring the acquisition of scientifically valid data.

### 2.3. Thioacetamide (TAA) Administration

Thioacetamide (TAA; CAS No. 62-55-5, ACS grade, Ph. Eur.) was obtained from Merck KGaA (Darmstadt, Germany). TAA was diluted in physiological saline solution and administered intraperitoneally to synanthropic rats at doses of 200, 210, 220, 230, 240, 250, 260, 270, 280, 290, and 300 mg/kg body weight. Injections were performed in the ventral region using a tuberculin syringe equipped with a 25 G × 16 mm needle.

TAA was administered intraperitoneally every three days over a total experimental period of 112 days, according to the predefined dosing schedule. Under this regimen, each animal received 37 consecutive injections.

This dosing strategy was designed to establish a chronic exposure model rather than an acute hepatotoxicity paradigm. Unlike short-term TAA protocols aimed at inducing rapid hepatocellular necrosis, the present administration schedule was structured to promote progressive and sustained hepatotoxic injury. The prolonged duration of treatment, repeated dosing intervals, and longitudinal monitoring of physiological, biochemical, hematological, and histopathological parameters collectively support the classification of this experimental design as a model of chronic TAA-induced hepatotoxicity.

In TAA-treated animals, respiratory rate, heart rate, and body weight were recorded every three days throughout the 112-day experimental period. This systematic monitoring allowed the evaluation of dose-dependent hepatotoxic effects based on physiological and systemic responses.

Once the sublethal dose associated with hepatotoxic manifestations was identified for each phenotypic variant, complementary hematological analyses were performed to assess systemic alterations related to progressive toxic exposure. This approach enabled controlled characterization of dose-dependent hepatotoxic responses under standardized experimental conditions.

### 2.4. Hematological and Biochemical Analysis

Hematological and biochemical parameters were evaluated in synanthropic rats receiving thioacetamide (TAA) doses associated with hepatotoxic effects. Blood samples were collected from the tail vein and transferred into capillary tubes containing EDTA for hematological analysis, as well as into tubes without anticoagulant for serum separation by centrifugation. Hematological and biochemical analyses were performed in a representative subset of animals per phenotypic variant (*n* = 3), selected at the median sublethal dose. This sample size was intentionally adopted due to the exploratory and model-establishment nature of the study, which aimed to characterize biological response patterns and the feasibility of a synanthropic rat model of TAA-induced hepatotoxicity, rather than to perform population-level statistical inference.

Hematological evaluation was primarily performed using an automated hematology analyzer (Genrui KT-40, Shenzhen Genrui Biotech Inc., Shenzhen, China) to determine total leukocyte counts and differential leukocyte profiles. Manual hematological procedures were additionally employed for confirmatory purposes and morphological assessment. For erythrocyte counting, blood samples were diluted 1:200 in Hayem’s solution (Chemquim E.I.R.Ltda., Lima, Peru) and quantified using a Neubauer chamber. Leukocyte counts were obtained following a 1:200 dilution in Turk’s solution (Chemquim E.I.R.Ltda., Lima, Peru). For differential leukocyte analysis, 10 µL of whole blood was smeared onto glass slides, air-dried at room temperature, and stained using Wright stain for 6 min, followed by phosphate buffer treatment (pH 6.4) for 4 min. Slides were examined under a light microscope using oil immersion at 100× magnification.

Serum biochemical analyses were performed using a semi-automatic chemistry analyzer URIT-880 (URIT Medical Electronic Co., Ltd., Guilin, China). The enzymatic activities of gamma-glutamyl transferase (GGT), aspartate aminotransferase (GOT), alanine aminotransferase (GPT), and alkaline phosphatase were measured, together with serum concentrations of albumin, glucose, and total proteins, employing commercial reagent kits (QCA Química Clínica Aplicada S.A., Amposta, Spain) according to the manufacturer’s instructions.

### 2.5. Histopathological Evaluation

Following euthanasia, a complete necropsy was performed, and liver tissues were excised for histopathological analysis. Excised livers were gently rinsed to remove residual blood and immediately fixed in 10% neutral buffered formalin for 24–48 h to preserve tissue architecture and prevent cellular degradation.

After fixation, liver samples were dehydrated through a graded series of ethanol solutions (70–100%), cleared in xylene, and embedded in paraffin using a tissue processor at 60 °C for 2 h. Paraffin-embedded tissues were sectioned at a thickness of 4–5 µm using a microtome. Tissue sections were mounted on glass slides and air-dried at room temperature for 24 h prior to staining.

Histological staining was performed using hematoxylin and eosin (H&E) following standard protocols. Histopathological alterations were evaluated by light microscopy and classified semiquantitatively according to their severity as mild, moderate, or severe. The degree of histological alteration was compared between TAA-treated groups and the control group.

### 2.6. Statistical Analysis

All statistical analyses and graphical representations, including box-and-whisker plots, were performed using R software version 4.3.2 (The R Foundation for Statistical Computing, Vienna, Austria). Data analysis was primarily descriptive, and graphical methods were used to visualize data distribution, variability, and biologically relevant trends across experimental groups.

Given the exploratory and model-establishment nature of the study, inferential statistical analyses were applied cautiously and interpreted in a supportive manner. Statistical significance was considered at a *p*-value ≤ 0.05. When applicable, data distribution and variance homogeneity were assessed prior to statistical comparisons to ensure the appropriateness of the applied analytical methods.

## 3. Results

### 3.1. Effects of TAA on Clinical and Physiological Parameters in Albino Synanthropic Rats

Clinical follow-up of albino synanthropic rats exposed to thioacetamide (TAA) revealed progressive physiological alterations across the evaluated dose range (200–300 mg/kg), affecting cardiovascular, respiratory, and body weight parameters.

**Figure 2:**
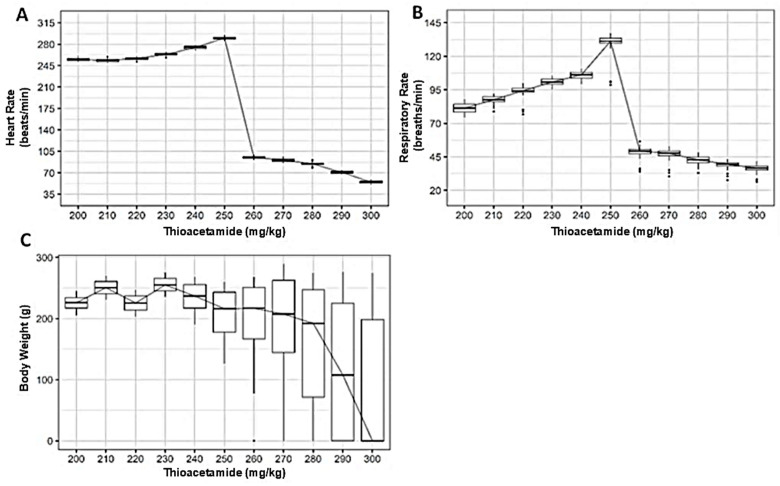
Dose-dependent effects of thioacetamide (TAA) on clinical and physiological parameters in albino synanthropic rats. Box-and-whisker plots show the effects of increasing intraperitoneal doses of TAA on heart rate (**A**), respiratory rate (**B**), and body weight (**C**). Data correspond to albino synanthropic rats receiving TAA every three days by intraperitoneal injection over a 12-week experimental period. The median value is represented by the horizontal line within each box.

Heart rate remained largely unchanged at lower TAA doses, with median values showing minimal variation between 200 and 240 mg/kg (Figure 2A). Beyond this range, a clear shift in cardiac response became evident. Rats receiving doses ≥ 260 mg/kg exhibited a marked reduction in heart rate, with median values declining progressively as TAA concentration increased.

Respiratory rate displayed a distinct response pattern (Figure 2B). At lower doses, respiratory frequency increased gradually, reaching maximal median values at approximately 250 mg/kg. This trend reversed abruptly at higher doses, where respiratory rates decreased sharply, particularly in animals exposed to ≥260 mg/kg.

Changes in body weight reflected the systemic impact of TAA exposure (Figure 2C). Animals treated with lower doses maintained relatively stable body weights throughout the experimental period. In contrast, higher TAA doses were associated with increased variability and a noticeable reduction in median body weight, most pronounced at doses ≥ 270 mg/kg.

Taken together, the clinical and physiological data indicate that albino synanthropic rats tolerate lower TAA doses without major systemic disruption, whereas higher concentrations are associated with marked alterations in vital parameters, consistent with progressive systemic involvement.

### 3.2. Dose-Dependent Effects of TAA on Vital Signs Across Synanthropic Rat Variants

**Figure 3:**
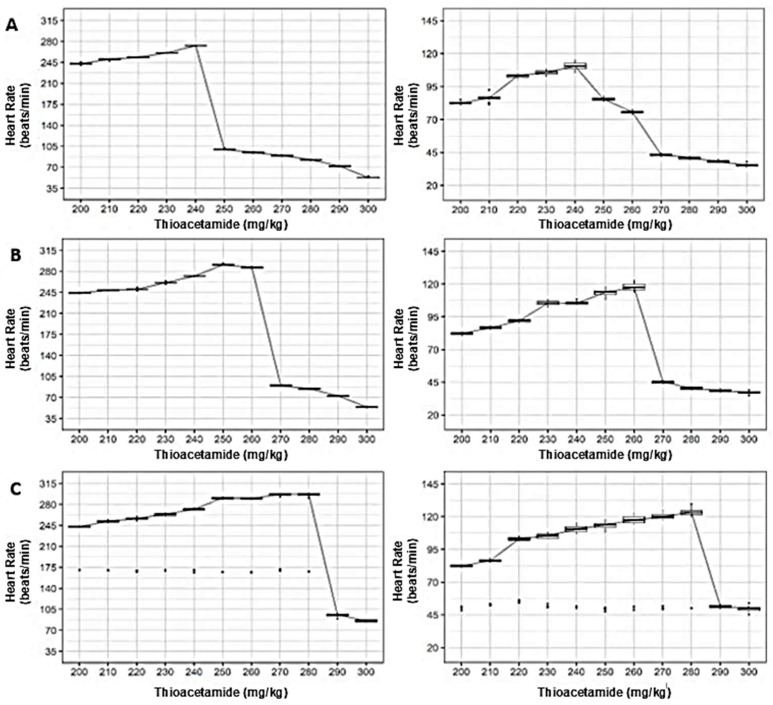
Dose-dependent effects of thioacetamide (TAA) on vital signs in synanthropic rat variants. Box-and-whisker plots show the effects of increasing intraperitoneal doses of TAA on heart rate and respiratory rate in mottled (**A**), black (**B**), and brown (**C**) synanthropic rats. Data correspond to animals receiving TAA every three days by intraperitoneal injection over a 12-week experimental period. The horizontal line within each box represents the median value.

When cardiovascular and respiratory responses were examined across mottled, black, and brown synanthropic rat variants, exposure to thioacetamide (TAA) revealed a shared dose-dependent pattern, albeit with phenotype-specific thresholds and response amplitudes (Figure 3).

In mottled rats, heart rate exhibited an initial upward modulation at lower TAA doses, followed by a pronounced decline at higher concentrations. Median heart rate values decreased sharply at doses ≥280 mg/kg, coinciding with a parallel suppression of respiratory rate after an initial gradual increase at lower exposure levels (Figure 3A).

Black synanthropic rats displayed a comparable response trajectory, although the transition from adaptive to suppressive physiological responses occurred at slightly lower doses. Heart rate increased up to intermediate concentrations before declining markedly at higher doses, while respiratory rate peaked at intermediate exposure levels and decreased substantially at doses ≥ 270 mg/kg (Figure 3B).

In brown rats, alterations in vital signs emerged earlier within the dose range. Both heart rate and respiratory rate increased across low and intermediate doses, followed by an abrupt reduction at higher concentrations, indicating a narrower tolerance window to increasing TAA exposure in this phenotype (Figure 3C).

### 3.3. Dose-Dependent Effects of TAA on Body Weight Across Synanthropic Rat Variant

**Figure 4:**
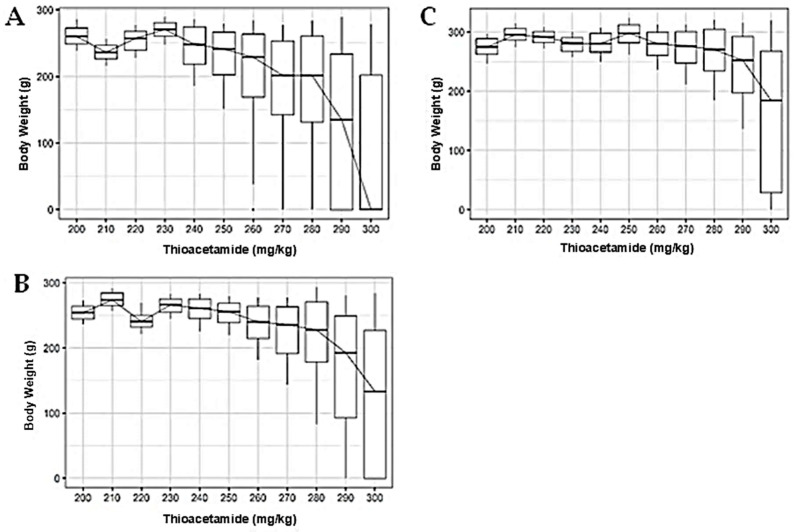
Dose-dependent effects of thioacetamide (TAA) on body weight in synanthropic rat variants. Box-and-whisker plots show changes in body weight in mottled (**A**), black (**B**), and brown (**C**) synanthropic rats following intraperitoneal administration of increasing doses of TAA. Animals received TAA every three days over a 12-week experimental period. The horizontal line within each box represents the median value.

Across mottled, black, and brown synanthropic rat variants, increasing exposure to thioacetamide (TAA) was associated with progressive alterations in body weight, which became more pronounced at higher doses (Figure 4).

At lower TAA concentrations (200–240 mg/kg), body weight remained largely stable in all evaluated phenotypes. As doses increased, a gradual reduction in median body weight emerged, accompanied by greater inter-individual variability. This effect was most evident at doses ≥ 270 mg/kg in mottled rats (Figure 4A) and at ≥280 mg/kg in black rats (Figure 4B).

Brown rats displayed a comparable response pattern, with relatively stable body weights at lower doses followed by a marked decline and increased dispersion at higher concentrations (Figure 4C). Overall, higher TAA exposure was consistently associated with loss of body mass across synanthropic rat variants, reflecting systemic involvement at elevated dose levels.

### 3.4. Effects of TAA on Serum Metabolic Parameters Across Synanthropic Rat Variants

**Figure 5:**
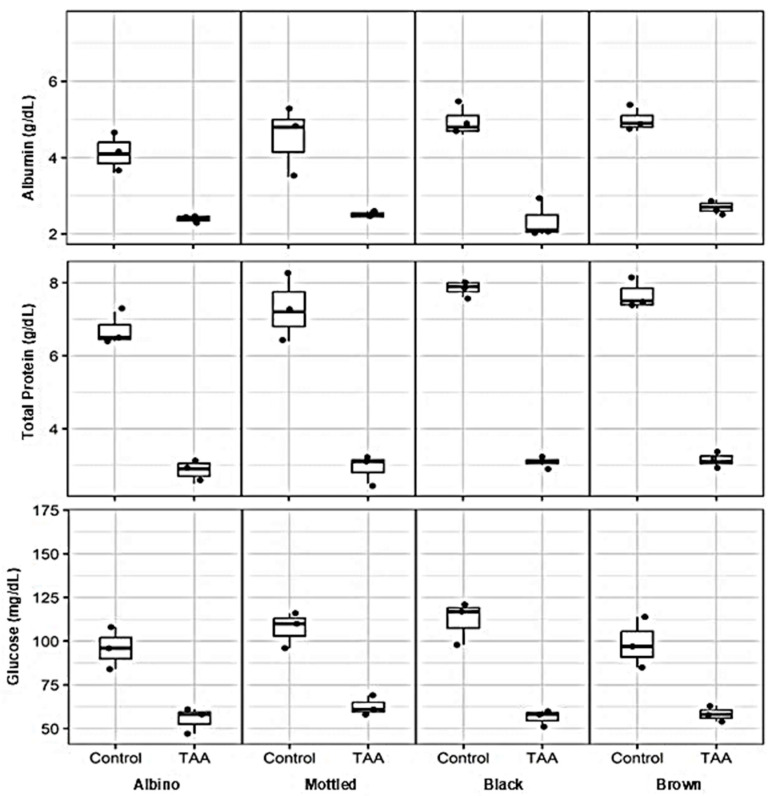
Effects of thioacetamide (TAA) on serum metabolic parameters in synanthropic rat variants. Box-and-whisker plots show serum concentrations of albumin, total proteins, and glucose in control and TAA-treated albino, mottled, black, and brown synanthropic rats. Animals received phenotype-specific intraperitoneal doses of TAA associated with hepatotoxic effects. Each data point represents an individual animal (*n* = 3 per group). The horizontal line within each box represents the median value.

Assessment of serum metabolic markers revealed clear alterations associated with hepatotoxic thioacetamide (TAA) exposure across all synanthropic rat variants when compared with their respective control groups (Figure 5). Changes were consistently observed in parameters related to hepatic synthetic function and systemic metabolic balance.

Serum albumin concentrations were markedly reduced in TAA-treated animals across all phenotypes. While control rats exhibited median albumin levels ranging from approximately 4.0 to 5.0 g/dL, exposure to hepatotoxic TAA doses resulted in substantially lower values, with median concentrations between 2.0 and 2.8 g/dL in albino, mottled, black, and brown rats.

A parallel reduction was observed in total serum protein levels. Control animals displayed median values between approximately 6.5 and 8.0 g/dL, whereas TAA-treated rats showed pronounced decreases, with median concentrations ranging from 2.8 to 3.5 g/dL across phenotypic variants.

Alterations in glucose homeostasis were also evident following TAA exposure. Control groups maintained median serum glucose levels between 90 and 120 mg/dL, while treated animals exhibited lower median values, typically ranging from 55 to 65 mg/dL. In some TAA-treated groups, glucose levels showed increased inter-individual variability.

### 3.5. Effects of TAA on Serum Liver Enzyme Activity Across Synanthropic Rat Variants

**Figure 6:**
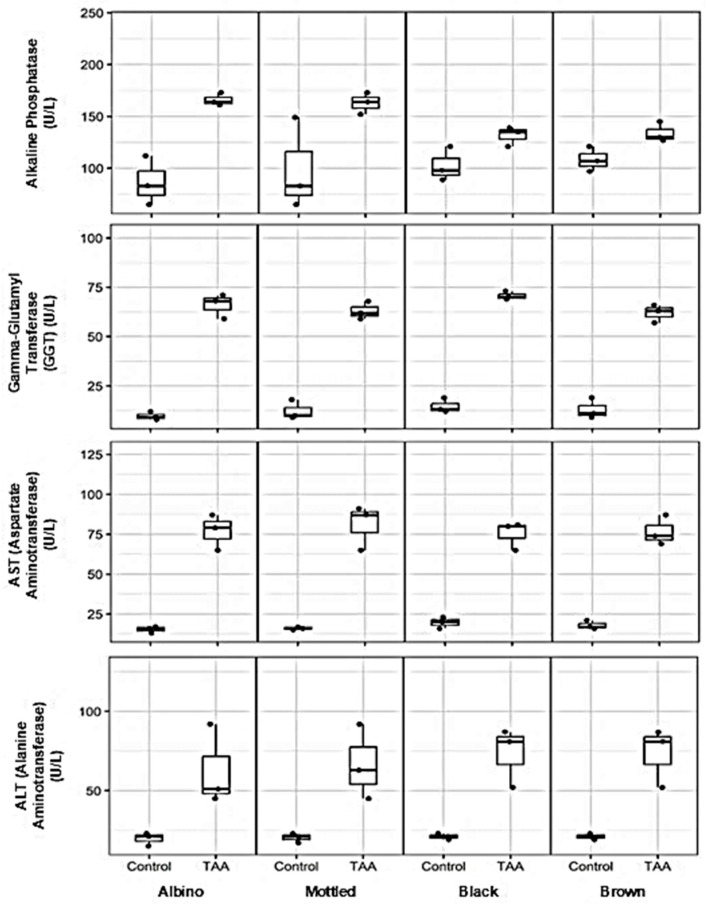
Effects of thioacetamide (TAA) on serum liver enzyme activity in synanthropic rat variants. Box-and-whisker plots show serum levels of alkaline phosphatase, gamma-glutamyl transferase (GGT), aspartate aminotransferase (AST/TGO), and alanine aminotransferase (ALT/TGP) in control and TAA-treated albino, mottled, black, and brown synanthropic rats. Animals received phenotype-specific intraperitoneal doses of TAA associated with hepatotoxic effects (albino: 250 mg/kg; mottled: 250 mg/kg; black: 270 mg/kg; brown: 290 mg/kg). Each data point represents an individual animal (*n* = 3 per group). The horizontal line within each box represents the median value.

Exposure to hepatotoxic doses of thioacetamide (TAA) resulted in pronounced alterations in serum liver enzyme activity across all synanthropic rat variants, consistent with hepatocellular injury and cholestatic involvement (Figure 6).

Markers associated with cholestatic dysfunction were markedly elevated following TAA administration. Serum alkaline phosphatase activity increased substantially in treated animals compared with controls, with median values rising from approximately 80–100 U/L to 130–170 U/L, depending on the phenotypic variant. A parallel pattern was observed for gamma-glutamyl transferase (GGT), whose median activity increased from baseline values of approximately 8–15 U/L in control rats to 60–75 U/L in TAA-treated animals across all variants.

Enzymes indicative of hepatocellular damage showed similarly robust responses. Serum aspartate aminotransferase (AST/TGO) activity increased from median values of approximately 15–25 U/L in control animals to 70–90 U/L following TAA exposure. Alanine aminotransferase (ALT/TGP) levels exhibited a comparable elevation, rising from approximately 18–25 U/L in controls to median values between 50 and 85 U/L in treated rats, with some degree of phenotypic variability.

### 3.6. Effects of TAA on Hematological Parameters Across Synanthropic Rat Variants

**Figure 7:**
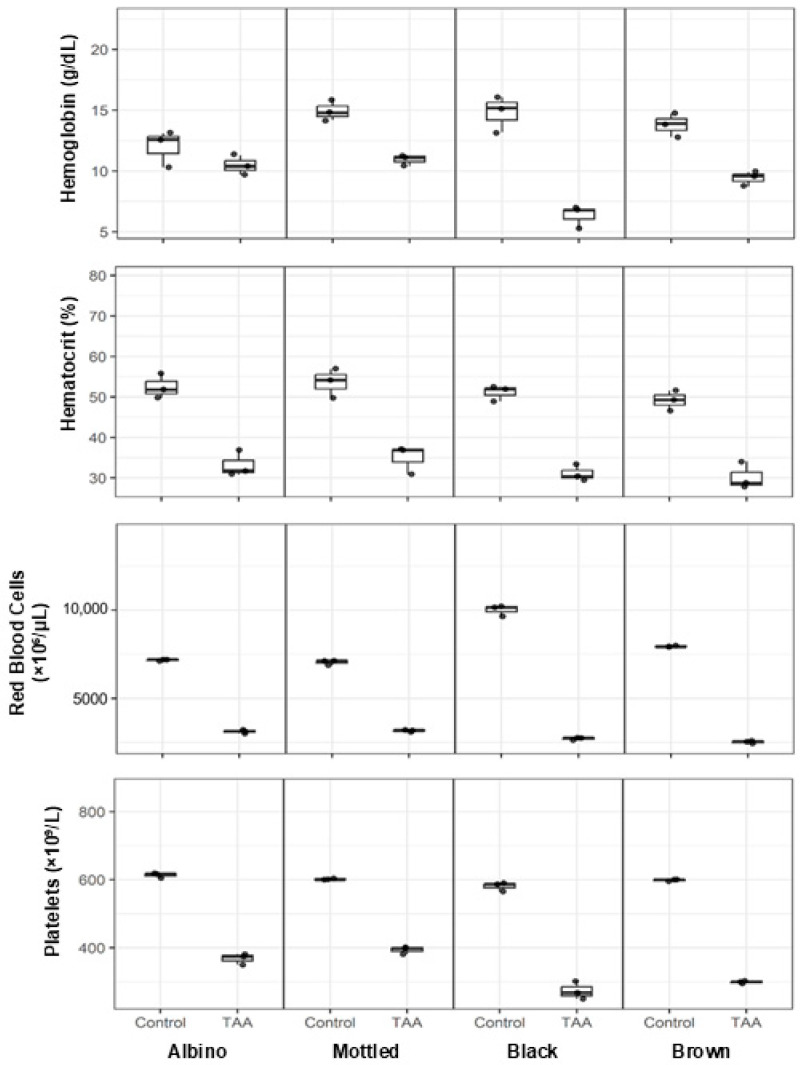
Effects of thioacetamide (TAA) on hematological parameters in synanthropic rat variants. Box-and-whisker plots show hemoglobin concentration, hematocrit percentage, erythrocyte count, and platelet count in control and TAA-treated albino, mottled, black, and brown synanthropic rats. Animals received phenotype-specific intraperitoneal doses of TAA associated with hepatotoxic effects (albino: 250 mg/kg; mottled: 250 mg/kg; black: 270 mg/kg; brown: 290 mg/kg). Each data point represents an individual animal (*n* = 3 per group). The horizontal line within each box represents the median value.

Hematological alterations were evident in synanthropic rats exposed to hepatotoxic doses of thioacetamide (TAA), affecting red blood cell-related parameters and platelet counts across all evaluated phenotypic variants (Figure 7).

Hemoglobin concentrations were consistently lower in TAA-treated animals compared with controls. Median values decreased from approximately 12–15 g/dL in control rats to 9–11 g/dL following TAA exposure, with more pronounced reductions observed in black and brown variants.

A parallel decrease was observed in hematocrit values. Control animals exhibited median hematocrit levels of approximately 48–55%, whereas TAA-treated rats showed reduced values ranging from 30% to 36% across phenotypes.

Erythrocyte counts were similarly affected. Median values declined from approximately 6800–10,000 × 10^3^/mm^3^ in control animals to 2500–3200 × 10^3^/mm^3^ in TAA-treated groups, depending on the variant.

Platelet counts were also reduced following TAA administration. While control rats displayed median platelet counts of approximately 580–620 × 10^9^/L, treated animals exhibited lower values, ranging from 270 to 400 × 10^9^/L across all synanthropic rat variants.

### 3.7. Effects of TAA on Leukocyte Count and Differential Across Synanthropic Rat Variants

**Figure 8:**
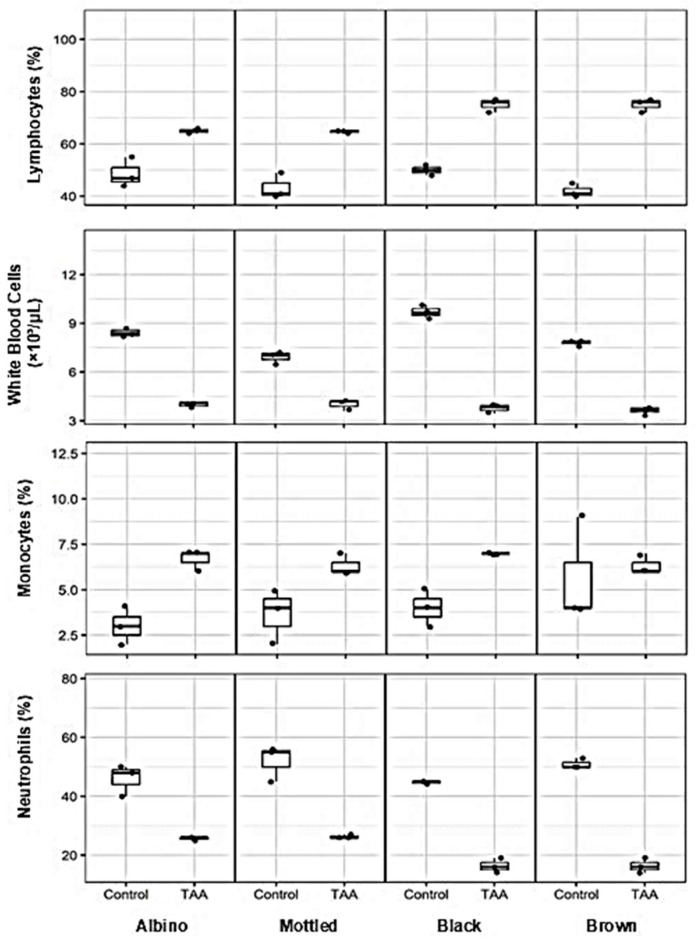
Effects of thioacetamide (TAA) on leukocyte count and differential leukocyte profiles in synanthropic rat variants. Box-and-whisker plots show total leukocyte count, lymphocyte percentage, monocyte percentage, and segmented neutrophil percentage in control and TAA-treated albino, mottled, black, and brown synanthropic rats. Animals received phenotype-specific intraperitoneal doses of TAA associated with hepatotoxic effects (albino: 250 mg/kg; mottled: 250 mg/kg; black: 270 mg/kg; brown: 290 mg/kg). Each data point represents an individual animal (*n* = 3 per group). The horizontal line within each box represents the median value.

Alterations in leukocyte counts and differential profiles were observed in synanthropic rats exposed to hepatotoxic doses of thioacetamide (TAA) across all evaluated phenotypic variants (Figure 8).

Total leukocyte counts were reduced in TAA-treated animals compared with controls. Median values decreased from approximately 7.0–10.0 × 10^3^ cells/mm^3^ in control rats to 3.0–4.0 × 10^3^ cells/mm^3^ following TAA exposure.

Differential analysis showed an increased proportion of lymphocytes in treated animals. Control groups exhibited median lymphocyte percentages of approximately 40–50%, whereas TAA-treated rats showed higher median values ranging from 60% to 75%, depending on the variant.

In contrast, the proportion of segmented neutrophils was markedly lower after TAA administration. Median values declined from approximately 45–55% in control animals to 15–25% in treated rats across all phenotypes.

Monocyte percentages exhibited a modest increase following TAA exposure, rising from median values of approximately 2–4% in control groups to 5–7% in TAA-treated animals.

### 3.8. Histopathological Features of the Liver in Control Synanthropic Rats

**Figure 9:**
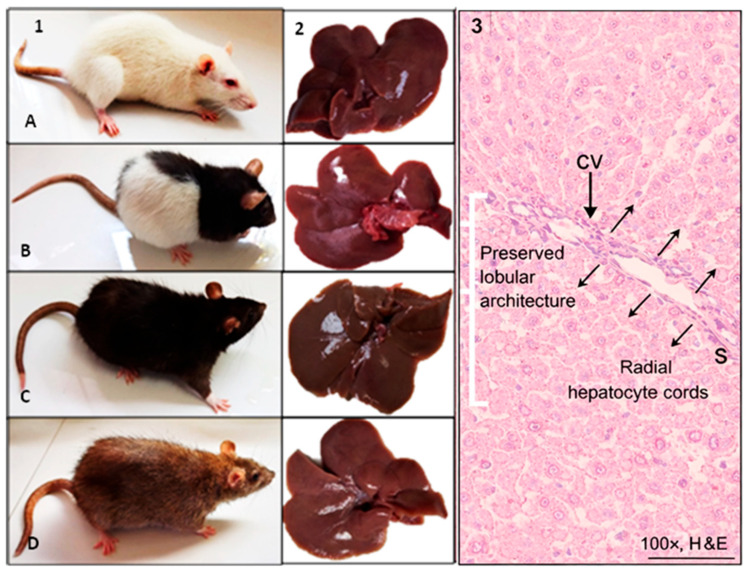
Gross and histological features of the liver in control synanthropic rats. Representative images of synanthropic rat phenotypic variants ((**1**); (**A**): albino, (**B**): mottled, (**C**): black, (**D**): brown) and corresponding liver specimens (**2**) from control animals not exposed to thioacetamide (TAA). Histological sections stained with hematoxylin and eosin (**3**) show preserved hepatic lobular architecture (bracket), with hepatocytes arranged in regular radial cords (arrows) surrounding a central vein (CV), and preserved sinusoidal spaces (S). No evidence of inflammatory infiltrates, necrosis, or architectural distortion is observed. Images shown are representative of the consistent histopathological features observed across animals within each control phenotype group. Original magnification 100×. Scale bar: 100 µm.

Gross anatomical examination of livers from control synanthropic rats revealed no visible abnormalities across all evaluated phenotypic variants (Figure 9). The hepatic lobes of albino, mottled, black, and brown rats displayed normal size, coloration, and surface appearance.

Histological analysis of hematoxylin and eosin-stained sections showed preserved hepatic architecture. Hepatocytes were arranged in regular radial cords surrounding a well-defined central vein, consistent with the typical lobular organization of normal liver tissue. Hepatocellular cytoplasm exhibited homogeneous eosinophilic staining with basophilic nuclei. No inflammatory infiltrates, cellular degeneration, necrosis, or architectural distortion were observed.

The micrographs presented in Figure 9 are representative of the consistent histopathological patterns observed across animals within each control phenotype group. Histological evaluation was performed systematically in all specimens following standardized fixation, tissue processing, sectioning, and staining procedures. The morphological features described in this section reflect recurrent and reproducible findings rather than isolated observations.

### 3.9. Gross and Histopathological Liver Alterations in TAA-Treated Synanthropic Rats

**Figure 10:**
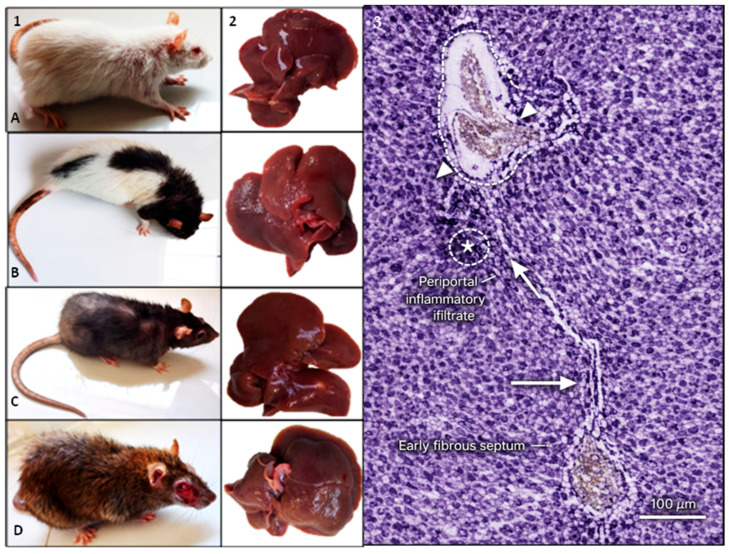
Clinical, gross, and histopathological liver alterations in TAA-treated synanthropic rats. Representative images of synanthropic rat phenotypic variants ((**1**); (**A**): albino, (**B**): mottled, (**C**): black, (**D**): brown) exposed to thioacetamide (TAA), showing clinical manifestations following hepatotoxic exposure. Corresponding liver specimens (**2**) display macroscopic alterations, including increased volume, irregular surface, pallor, vascular congestion, and nodular appearance. Histological sections stained with hematoxylin and eosin (**3**) show centrilobular hepatocellular necrosis, periportal inflammatory infiltrates (*), cytoplasmic vacuolization, increased apoptotic bodies, and expansion of fibrous tissue within the hepatic parenchyma, including portal fibrosis (dashed outline), expansion of the portal area (arrowheads), and early fibrous septum formation (arrows). Images shown are representative of the consistent clinical and histopathological alterations observed across animals within each TAA-treated phenotype group. Scale bar: 100 µm.

Synanthropic rats exposed to phenotype-specific hepatotoxic doses of thioacetamide (TAA) developed evident clinical deterioration accompanied by marked macroscopic and histopathological liver alterations (Figure 10).

Clinically, TAA-treated animals exhibited reduced spontaneous activity, altered exploratory behavior, roughened fur, and a visible decline in overall body condition. These manifestations were consistently observed across albino, mottled, black, and brown variants following sustained TAA exposure.

Gross examination of liver specimens revealed pronounced morphological changes. Hepatic enlargement was frequently observed, along with irregular surface contours, areas of pallor, and vascular congestion. In several specimens, nodular structures were evident on the liver surface, indicating advanced tissue remodeling in response to chronic hepatotoxic insult.

Histological evaluation of hematoxylin and eosin-stained sections demonstrated extensive disruption of hepatic architecture. Centrilobular hepatocellular necrosis was a prominent feature, accompanied by periportal inflammatory infiltrates and cytoplasmic vacuolization. Increased numbers of apoptotic bodies were observed within the parenchyma, together with expansion of fibrous tissue and loss of normal lobular organization. These alterations were consistently present across all synanthropic rat variants exposed to TAA.

In addition to acute hepatocellular injury, histopathological examination revealed clear evidence of fibrotic remodeling in TAA-treated animals. Periportal fibrous expansion, formation of fibrous septa bridging adjacent vascular structures, and architectural distortion of the hepatic parenchyma were consistently observed across phenotypic variants. These structural alterations indicate progression beyond acute toxic injury and are compatible with chronic hepatotoxic exposure. Although formal quantitative fibrosis staging was not performed, the morphological features observed support the presence of established fibrotic changes in this model.

The histological images shown in Figure 10 are representative of the consistent morphological alterations observed across animals within each TAA-treated phenotype group. All specimens were evaluated under identical histological conditions, and the features reported in this section correspond to reproducible patterns identified throughout the experimental groups rather than isolated findings.

## 4. Discussion

The present study demonstrates that synanthropic rats develop marked hepatotoxic and fibrosis-like alterations following experimental induction with thioacetamide (TAA), supporting their potential use as an alternative preclinical model in hepatology and toxicology research. The biochemical, hematological, and histopathological changes observed are consistent with those described in classical laboratory rat strains exposed to TAA, reinforcing the robustness and reproducibility of this chemically induced liver injury model [5,7].

The use of TAA as the sole hepatotoxic agent was intentional, as the primary objective of this study was to validate its hepatotoxic effects in synanthropic rats rather than to conduct comparative toxicological analyses across multiple compounds. Employing a well-characterized and extensively documented hepatotoxic agent enabled focused assessment of susceptibility and injury patterns within this genetically heterogeneous population.

Although cardiovascular parameters were recorded during the study, their evaluation was incorporated as part of systemic physiological monitoring rather than as a primary endpoint focused on cardiac toxicity. The central objective and interpretation of findings remained directed toward hepatic injury and liver-specific toxicological alterations.

Administration of TAA in synanthropic rats resulted in characteristic alterations associated with hepatocellular injury, including significant elevations in serum transaminases, gamma-glutamyl transferase, and alkaline phosphatase, together with systemic hematological disturbances and structural liver damage. Similar biochemical profiles have been reported in Wistar and Sprague–Dawley rats, where enzyme elevations reflect hepatocellular necrosis, cholestasis, and impaired hepatic function following TAA exposure [5,7].

Histopathological examination revealed centrilobular necrosis, periportal inflammatory infiltrates, cytoplasmic vacuolization, and disruption of hepatic architecture. These findings are consistent with previous studies demonstrating that prolonged TAA exposure induces structural remodeling consistent with chronic liver injury.

While classical histopathological evaluation revealed pronounced centrilobular necrosis and the presence of apoptotic bodies consistent with TAA-induced hepatocellular injury, we acknowledge that apoptosis-specific assays such as TUNEL staining or cleaved caspase-3 immunohistochemistry could provide additional mechanistic resolution. Hepatocellular injury in toxic liver models does not uniformly correspond to apoptosis-dominant mechanisms; indeed, substantial liver damage may occur in the relative absence of caspase-mediated apoptosis depending on the injury context [17]. In TAA-induced liver injury, apoptosis markers have been incorporated primarily in mechanistic studies aimed at dissecting signaling pathways involved in fibrosis progression and inflammatory modulation [18]. Nevertheless, classical morphological criteria—including centrilobular necrosis, cytoplasmic vacuolization, inflammatory infiltration, and architectural disruption—remain widely accepted indicators of hepatocellular damage in chemically induced liver injury models [19,20]. Future studies incorporating apoptosis-specific molecular approaches may further delineate the balance between necrotic and apoptotic pathways in synanthropic rat models of hepatotoxicity.

In addition, although lipid-specific staining such as Oil Red O is widely used to detect neutral lipid accumulation in steatotic or metabolic liver disease models, thioacetamide (TAA) is classically characterized as a hepatotoxic agent that induces liver injury predominantly through oxidative stress, centrilobular necrosis, inflammatory infiltration, and progressive fibrotic remodeling rather than primary lipid-driven steatosis. Experimental studies have consistently demonstrated that TAA bioactivation generates reactive metabolites leading to hepatocellular injury and inflammation, with fibrosis development in chronic exposure settings, but without dominant macrovesicular steatosis as a defining feature [17,18]. In the present study, histopathological evaluation revealed centrilobular necrosis, architectural distortion, vascular congestion, inflammatory infiltration, and connective tissue expansion across TAA-treated groups, findings consistent with the classical toxicological profile of TAA-induced injury. No predominant morphological patterns suggestive of lipid-driven hepatopathy were observed. Therefore, while Oil Red O staining may be informative in models specifically targeting metabolic dysregulation or steatosis, it was not incorporated into the current experimental design, which focused on establishing and characterizing hepatotoxic injury patterns in synanthropic rats. Future investigations aimed at exploring metabolic remodeling under environmentally heterogeneous conditions may benefit from complementary lipid-specific analyses.

When compared with other hepatotoxins, TAA exhibits distinct injury profiles. Acetaminophen predominantly induces acute hepatocellular necrosis associated with rapid transaminase elevation and glutathione depletion, whereas carbon tetrachloride (CCl_4_) and TAA are widely employed to induce progressive liver injury and fibrotic remodeling following repeated exposure. These distinctions underscore the suitability of TAA for modeling chronic hepatotoxicity and fibrotic changes.

An important contribution of this study lies in the use of synanthropic rats, which exhibit greater genetic heterogeneity and environmental exposure history compared to conventional laboratory strains. This variability may result in physiological responses that more closely resemble the heterogeneity observed in human liver disease. Previous studies have suggested that non-inbred or wild-derived rodents display broader endocrine and metabolic profiles, potentially influencing susceptibility and response to hepatotoxic insults [3].

The present findings should be interpreted within the broader discussion on model representativeness and translational applicability. Although conventional inbred rat strains are widely used for hepatotoxicity research, the use of a genetically heterogeneous synanthropic population allowed us to explore whether classical TAA-induced injury patterns are maintained under conditions of increased biological variability. The consistent biochemical, hematological, and histopathological alterations observed in this study suggest that the fundamental toxicodynamic mechanisms of TAA remain robust across distinct phenotypic backgrounds. These results align with ongoing discussions in translational science emphasizing that experimental robustness should ideally be evaluated across varying biological contexts rather than exclusively within highly standardized conditions [1,3]. By demonstrating reproducible hepatotoxic injury in a heterogeneous population, our findings support the concept that classical toxicological paradigms can extend beyond conventional inbred strains, providing complementary translational insight.

Nevertheless, increased biological variability also represents a limitation. Nutritional status, environmental history, and individual physiological differences can modulate susceptibility to TAA-induced liver injury. Although housing and dietary conditions were standardized during the acclimatization period, intrinsic biological variability among synanthropic rats remains an inherent characteristic of the model.

Only female animals were included in this study to maintain experimental uniformity under controlled housing conditions. Sex-based differences in hepatic metabolism and susceptibility to toxic injury have been documented in rodent models, particularly in relation to cytochrome P450 activity and inflammatory signaling pathways. The exclusive use of females reduced hormonal and metabolic variability, thereby strengthening internal consistency; however, extrapolation to male subjects warrants further investigation.

Although Sirius Red staining is widely recognized as a sensitive method for detecting and quantifying collagen deposition in chronic liver injury models, the present study was primarily designed to characterize systemic hepatotoxic responses to thioacetamide exposure rather than to establish an advanced fibrosis quantification model. Histopathological evaluation using hematoxylin and eosin staining revealed consistent architectural disruption, inflammatory infiltration, hepatocellular necrosis, and fibrotic expansion across treated groups. Future investigations specifically focused on fibrosis progression and quantitative collagen assessment in synanthropic rat populations may benefit from incorporating Sirius Red staining and morphometric fibrosis analysis.

Overall, the biochemical and morphological alterations observed in this model align with previous reports describing reproducible hepatotoxicity induced by TAA [2]. Taken together, these findings support the use of synanthropic rats as a viable alternative experimental platform for hepatotoxicity studies, offering potential advantages in ecological relevance and translational applicability when appropriately controlled.

## 5. Conclusions

This study establishes a preclinical model of chemically induced hepatotoxicity in synanthropic rats using controlled administration of thioacetamide (TAA). Exposure to TAA resulted in consistent hematological, biochemical, histopathological, and behavioral alterations compatible with acute and subchronic liver injury.

The observed responses showed substantial concordance with those reported in conventional laboratory rat models, supporting the use of synanthropic rats as a viable alternative for experimental hepatotoxicity and pharmacological research. Despite their genetic and environmental heterogeneity, synanthropic rats exhibited reproducible patterns of liver injury, which may enhance the ecological and translational relevance of preclinical studies.

Future investigations should focus on deeper genetic and metabolic characterization of synanthropic rat populations and on further standardization of experimental conditions to reduce interindividual variability. The incorporation of such alternative models may strengthen toxicological research and contribute to more robust assessments of hepatic safety in preclinical settings.

## Data Availability

The original contributions presented in this study are included in the article. Further inquiries can be directed to the corresponding author.
